# How accurate is MRI for diagnosing tarsal coalitions? A retrospective diagnostic accuracy study

**DOI:** 10.1007/s00330-023-10304-z

**Published:** 2023-10-19

**Authors:** Adrian A. Marth, Georg C. Feuerriegel, Roy P. Marcus, Reto Sutter

**Affiliations:** 1 Department of Radiology, Balgrist University Hospital, Faculty of Medicine, University of Zurich, Forchstrasse 340, 8008 Zurich, Switzerland; 2 Swiss Center for Musculoskeletal Imaging, Balgrist Campus AG, Zurich, Switzerland

**Keywords:** MRI, Tarsal bones, Tarsal coalition, Data accuracy

## Abstract

**Objectives:**

This study aimed to evaluate the diagnostic accuracy, inter-reader agreement, and associated pathologies on MR images of patients with confirmed TC.

**Methods and materials:**

In this retrospective study, 168 ankle MRI exams were included, consisting of 56 patients with clinically or surgically confirmed TC and 112 controls without TC, matched for age and sex. Images were analyzed independently by three radiologists blinded to clinical information. The evaluation criteria included the presence, type, and location of TC, as well as associated pathologies. After calculating diagnostic accuracy and the odds ratio of demographic data and anatomic coalition type for associated pathologies, inter-reader agreement was assessed using kappa statistics.

**Results:**

The majority of TCs were non-osseous (91.1%) and located at the calcaneonavicular (33.9%) or talocalcaneal joint (66.1%). Associated pathologies included adjacent and distant bone marrow edema (57.1% and 25.0%), osteochondral defect of the talar dome (OCD, 19.6%), and joint effusion (10.7%) and accessory anterolateral talar facet (17.9%). Talar OCD was associated with increased patient age (*p* = 0.03). MRI exhibited a cumulative sensitivity and specificity of 95.8% and 94.3% with almost perfect inter-reader agreement (*κ* = 0.895).

**Conclusion:**

MRI is a reliable method for detecting tarsal coalition and identifying commonly associated pathologies. Therefore, we recommend the routine use of MRI in the diagnostic workup of patients with foot pain and suspected tarsal coalition.

**Clinical relevance statement:**

MRI is an accurate and reliable modality for diagnosing tarsal coalitions and detecting associated pathologies, while improving patient safety compared to computed tomography by avoiding radiation exposure.

**Key Points:**

*• Despite the technological progress in magnetic resonance imaging (MRI), computed tomography (CT) is still regarded as the gold standard for diagnosing tarsal coalition (TC).*

*• MRI had a cumulative sensitivity of 95.8% and specificity of 94.3% for detecting tarsal coalition with an almost perfect inter-reader agreement.*

*• MRI demonstrates high accuracy and reliability in diagnosing tarsal coalitions and is useful for identifying associated pathologies, while also improving patient safety by avoiding radiation exposure.*

## Introduction

Tarsal coalitions (TCs) are a congenital condition caused by a deficient embryologic bone segmentation leading to an abnormal union between two or more tarsal bones [1]. The reported incidence ranges from 1 to 14% in the general population, although these numbers are uncertain because only 25% of all tarsal coalitions become symptomatic [2–4]. Bilateral coalitions are reported in 50% of all cases and the most common anatomic locations are the talocalcaneal and calcaneonavicular joints, accounting for 90% of all coalitions [2, 5].

Histologically, tarsal coalitions consist of either an osseous or non-osseous (fibrous or cartilaginous) bone bridging. While the majority of calcaneonavicular coalitions are non-osseous, three different evenly distributed histologic subtypes are found in talocalcaneal coalitions (34% osseous, 28% cartilaginous, 39% fibrous) [6]. Patients with tarsal coalition typically present with hindfoot pain and stiffness with decreased subtalar motion, valgus deformity, or ankle sprains [7, 8].

Conservative treatment is the first-line therapy consisting of foot arch support, cast immobilization, and anti-inflammatory medication [6], while surgical treatment is reserved for therapy-refractory cases, consisting of bridge resection with an interposition of soft tissue structures or primary arthrodesis [9].

Initially, patients with suspected tarsal coalition typically undergo conventional radiography. However, it is recognized that especially talocalcaneal and non-osseous coalitions are difficult to diagnose due to bone overlap or coalition obliquity, limiting the assessment to secondary signs suggestive of TC. The sensitivity of those secondary signs has a great variability ranging from 48 to 88% [10]. Thus, patients often undergo cross-sectional imaging for definitive TC assessment [11]. Computed tomography (CT) has generally been accepted as the gold standard for TC assessment due to high sensitivity and specificity, its ability to depict the complex anatomy of the hindfoot articulations, and concomitant usefulness in preoperative planning [12–16]. On the other hand, some authors advocate the usefulness of magnetic resonance imaging (MRI) to visualize fibrous or cartilaginous tissue, bone stress response adjacent to the TC, or soft tissue abnormalities [8, 17, 18]. This topic is of growing importance because recently, several authors reported a suspected association of TC with osteochondral defects of the talar dome (OCD) and bone stress injuries of the mid- and hindfoot [19, 20]. Furthermore, there appears to be an association with an accessory anterolateral talar facet (AALTF), which is a developmental bone segmentation defect characterized by squaring of the apex of the lateral talar aspect, resulting in a contact with the angle of Gissanne [21, 22]. However, only a few studies with small sample sizes conducted in the 1990s and one small case series conducted in the 2000s have investigated the diagnostic performance of MRI in detecting TC [23–26]. Therefore, the aim of this study was to demonstrate the diagnostic performance of MRI in the detection of TC and to evaluate associated pathologies of the hindfoot.

## Material and methods

### Study design and patient selection

This retrospective study was approved by the local ethics committee (approval No. 2023–00491) and the need for written informed consent was waived. All procedures were conducted in compliance with the ethical standards set by the institutional and/or national research committee, as well as in accordance with the 1964 Helsinki Declaration and its subsequent amendments, or other equivalent ethical standards.

The hospital’s radiology information system was queried for all performed MRI examinations of the ankle and the term “coalition” between January 2013 and December 2022. The recorded diagnosis of TC was made by podiatric surgeons with the following clinical workflow: First, a detailed history of patients admitted for foot pain was conducted by the surgeon, which included inquiring about complaints of foot pain, duration of symptoms, and any relevant family history of foot problems. A physical examination of the affected foot was then performed, including assessment of range of motion, foot stability, and palpation for areas of tenderness or bony prominence. During the clinical evaluation, the surgeon also specifically examined common clinical signs of tarsal coalition, including rigid flatfoot deformity, limited or painful foot motion, and lack of foot positional flexibility. Next, imaging was performed to support the suspected clinical diagnosis. The first step was to obtain radiographs, including anteroposterior, lateral, and oblique views. After obtaining radiographs for initial imaging assessment, cross-sectional MRI was performed if radiographs were inconclusive. Patients were included in the study only if history, clinical examination, and imaging were consistent. In the next step, incomplete examinations (one or more missing sequences from the standard clinical protocol stored in the local Picture Archiving and Communications System (PACS)) and patients with history of ankle or foot surgery were excluded. In order to evaluate the diagnostic performance, a control group undergoing MRI examinations of the ankle between January 2013 and December 2022 was included and matched by age, sex, and MRI field strength to the study group. Exclusion criteria of the control group included (1) evidence of TC on MRI or other imaging modalities in the local PACS, (2) clinical or surgical report diagnosing TC, (3) incomplete MRI examinations, and (4) history of ankle or foot surgery.

### Image analysis

MR examinations were performed on 1.5-T and 3.0-T scanners (MAGNETOM Avanto^fit^ and MAGNETOM Skyra^fit^, Siemens Healthineers) and consisted of the following sequences: sagittal short tau inversion recovery (STIR), coronal proton density (PD)-weighted in-phase and water-only DIXON, axial PD-weighted water-only DIXON, and T1-weighted oblique axial sequence, with detailed sequence parameters described in Table 1. The retrospective analysis was performed by three radiologists (A.A.M., G.C.F., R.P.M.) with 4, 5, and 9 years of experience in musculoskeletal radiology. Prior to reading, a training session was conducted to familiarize readers with the study objectives and scoring guidelines. Readers were blinded to demographic and clinical data and analyzed the images in random order on the local PACS viewer (Merlin, Phoenix PACS). None of the readers was involved in the selection process of the TC group or the control group. All readers were instructed to characterize the anatomic location and type of TC, if present. Bone marrow edema was characterized by bone marrow signal intensity changes observed on STIR or water-only DIXON sequences, while joint effusion was defined as an abnormal accumulation of fluid in the joint space of the tibiotalar, subtalar, or midtarsal joint. OCD was identified if a focal defect and/or detachment of the cartilage and underlying subchondral bone in the ankle joint was visible. The presence of an accessory anterolateral talar facet (AALTF) was evaluated on sagittal STIR images. If bone marrow edema was present adjacent to the TC, this was further specified as “adjacent bone marrow edema” (within the margins of < 2 cm surrounding the TC) or as “distant bone marrow edema” (> 2 cm distance from TC). For the control group, the presence of bone marrow edema, talar OCD, and joint effusion was recorded.

**Table 1:** Sequence parameters for the clinical protocol of 1.5-T and 3.0-T MRI scans. *PD*, proton density; *STIR*, short tau inversion recovery; *TSE*, turbo spin echo

	STIR (sagittal)	PD TSE Dixon (coronal)	PD TSE Dixon (axial)	T1 TSE (oblique axial)
Repetition time (ms)	4000	3700	4350	430
Time to echo (ms)	30	42	42	13
Inversion time (ms)	150	-	-	-
Field of view	270 × 270	160 × 160	160 × 160	160 × 160
Matrix size	512 × 512	358 × 512	358 × 512	358 × 512
Slice thickness (mm)	3.0	3.0	4.0	4.0
Acquisition time	2′24″	4′30″	3′40″	1′20″

### Statistical analysis

All statistical analyses were performed using SPSS Statistics (v25, IBM Corp.). Sensitivity and specificity of each reader were analyzed using cross tables. Cumulated sensitivity and specificity were defined as the average sensitivity and specificity of all three readers [27]. Moreover, subgroup analyses for diagnostic accuracy were performed for different MRI field strengths. Inter-reader agreement was assessed with a kappa statistic (Fleiss’ kappa) and level of agreement was reported according to Landis et al (≥ 0.8: almost perfect; 0.61–0.8: substantial; 0.41–0.6: moderate; 0.21–0.4: fair; 0.2–0.01: slight, ≤ 0: poor) [28]. Confirmation of normal distribution and homogeneity of variances for continuous variables was achieved by the Kolmogorov–Smirnov test and Levene test. Group differences in patients with associated imaging pathologies were analyzed with a chi-squared analysis for categorical and Student’s* t* test for continuous variables. The odds of associated pathologies to patient age, sex, and anatomic location of TC were computed using a binary logistic regression. Because most coalitions were non-osseous, the influence of histologic subtype was not further statistically analyzed. Statistical significance was assumed for an alpha level < 0.05.

## Results

### Location and type of tarsal coalitions (TCs)

**Fig. 1:**
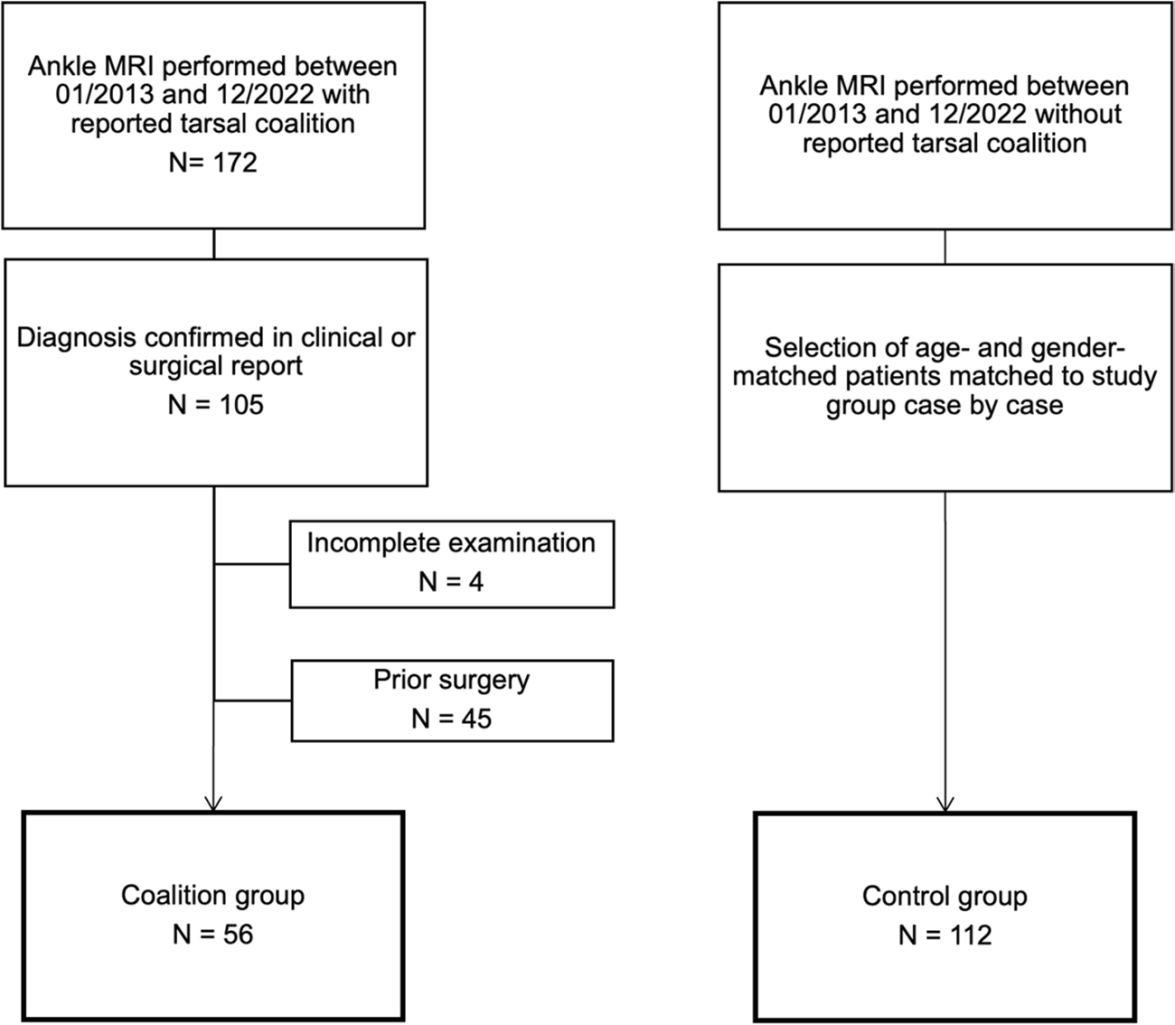
Flow chart depicting the case selection process

A total of 56 patients were identified for the coalition group and 112 cases for the control group (Fig. 1).

In the coalition group, a total of 23 cases (41.1%) reported a history of trauma, compared to 51 cases (45.5%) in the control group (*p* = 0.863). Mean age of the 56 patients with TC (*n* = 56) was 27.5 ± 13.3 years (46.4% female), whereas the mean age of the 112 patients in the control group was 27.6 ± 14.1 years (45.5% female). TC was confirmed intraoperatively in 36 (64.2%) and clinically in 20 (35.7%) cases. There was no significant difference between age and sex for the coalition group and the control group (*p* ≥ 0.398; Table 2). Coalitions were mainly of the non-osseous type (91.1%) and were present either in calcaneonavicular location (33.9%; 100% non-osseous) or in talocalcaneal location (66.1%; 86.5% non-osseous). Talocalcaneal coalitions were located either in the middle facet (10.7%), in the middle and posterior facet (50.0%), or in the posterior facet only (5.3%).

**Table 2:** Coalition and control group characteristics. *OCD*, osteochondral defect

	Coalition group (*n* = 56)	Control group (*n* = 112)	*p*-value
Age (years)	27.5 ± 13.3	27.6 ± 14.1	0.921
Sex (female; *n*, %)	26 (46.4)	51 (45.5)	0.698
Coalition type (*n*, %)			
Osseous coalition	5 (8.9)		
Non-osseous coalition	51 (91.1)		
Coalition location (*n*, %)			
Talocalcaneal coalition	37 (66.1)		
* Middle facet*	6 (10.7)		
* Middle and posterior facet*	28 (50.0)		
* Posterior facet*	3 (5.4)		
Calcaneonavicular coalition	19 (33.9)		
Associated pathologies (*n*, %)			
Bone marrow edema	46 (82.1)		
* Adjacent*	32 (57.1)	72 (64.3)	0.170
* Distant*	14 (25.0)		
Talar OCD	11 (19.6)	10 (8.9)	0.047*
* Medial talar dome*	9 (16.1)		
* Lateral talar dome*	1 (1.8)		
Joint effusion	6 (10.7)	20 (17.9)	0.228
Accessory anterolateral facet	10 (17.9)	8 (7.1)	0.039*

^*^ denotes statistical significance

### Sensitivity and specificity and inter-reader agreement for diagnosing TC

MRI of patients with TC was performed at 1.5 T in 18 cases (32.1%) and at 3.0 T in 38 cases (67.9%), while MRI examinations of the control group were performed at 1.5 T in 32 cases (28.6%) and 3.0 T in 80 cases (71.4%, *p* = 0.557). Cumulated sensitivity and specificity to diagnose TC were 95.8% and 94.3%, respectively. The overall individual sensitivity and specificity as well as for different MRI field strengths for each reader are reported in Table 3. Among individual readers, the minimum overall observed sensitivity was 94.6% and the minimum specificity was 92.8%. For the 1.5-T MRI subgroup, the minimum sensitivity observed was 94.4%, and the minimum specificity was 87.5%, while for 3-T MRI, the minimum sensitivity was 94.7% and the minimum specificity was 95.0%.

**Table 3:** Overall individual sensitivity and specificity for each reader and at different MRI field strengths for diagnosing tarsal coalitions. *CI*, confidence interval; *MRI*, magnetic resonance imaging

	Sensitivity (95% CI)	Specificity (95% CI)
Overall		
Reader 1	53/56 (0.946; 0.851–0.988)	107/112 (0.955; 0.884–0.986)
Reader 2	54/56 (0.964; 0.874–0.997)	106/112 (0.946; 0.869–0.978)
Reader 3	54/56 (0.964; 0.874–0.997)	104/112 (0.928; 0.841–0.963)
1.5-Tesla MRI		
Reader 1	17/18 (0.944; 0.724–1.000)	29/32 (0.906; 0.750–0.975)
Reader 2	17/18 (0.944; 0.724–1.000)	29/32 (0.906; 0.750–0.975)
Reader 3	17/18 (0.944; 0.724–1.000)	28/32 (0.875; 0.713–0.956)
3-Tesla MRI		
Reader 1	36/38 (0.947; 0.818–0.995)	78/80 (0.975; 0.908–0.998)
Reader 2	37/38 (0.974; 0.853–1.000)	77/80 (0.963; 0.891–0.992)
Reader 3	37/38 (0.974; 0.853–1.000)	76/80 (0.950; 0.875–0.984)

**Fig. 2:**
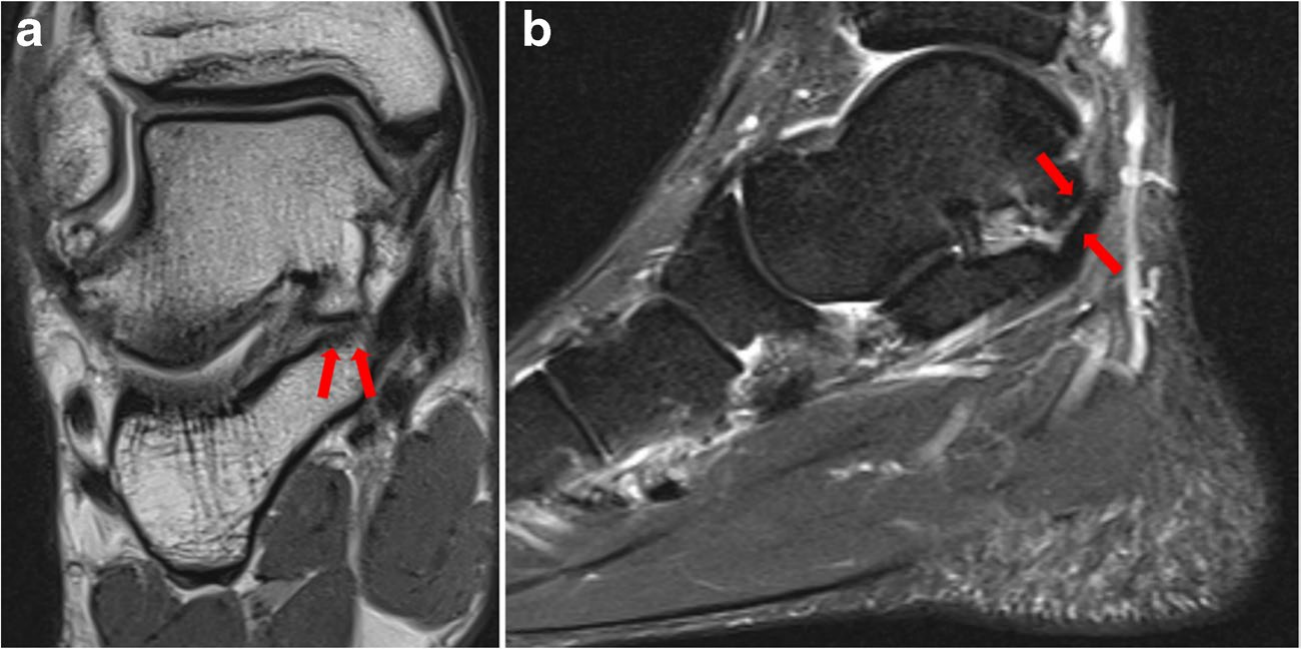
Coronal PD-weighted DIXON image and sagittal STIR image of a talocalcaneal coalition in posterior location (arrows) in a 28-year-old male patient admitted for pain in the left foot (**a, b**). The coalition in this location was missed by all three readers

**Fig. 3:**
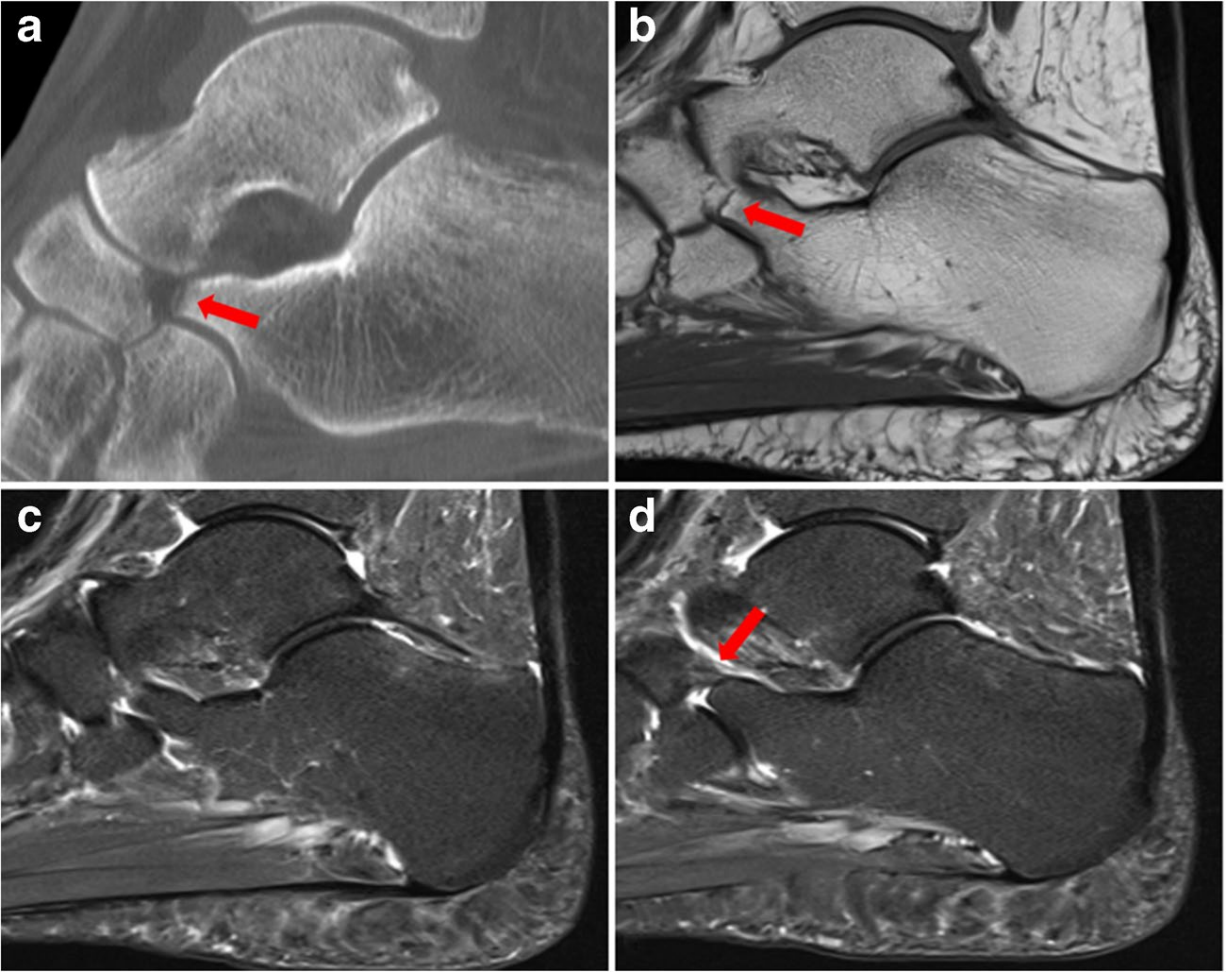
33-year-old patient of the control group with a history of right ankle trauma, which was the single case that was false-positive for two out of the three readers. The patient had been diagnosed with an avulsion fracture of the calcaneal tip (arrow) on computed tomography (**a**). The MR examination acquired 4 months later depicting cortical irregularity of the calcaneonavicular junction (arrow) on sagittal T1-weighted image (**b**) with a false-positive diagnosis of a calcaneonavicular coalition for two readers. Two adjacent sagittal STIR images in the same patient reveal a thickening of the calcaneonavicular band of the bifurcate ligament (arrow), which was mistaken for a coalition by two readers (**c**,** d**)

Overall inter-reader agreement was almost perfect (*κ* = 0.895, 95% confidence interval (CI) 0.801–0.990). This also applied for talocalcaneal coalitions (*κ* = 0.883 (95% CI 0.789–0.978)) and calcaneonavicular coalitions (*κ* = 0.941 (95% CI 0.847–1.035)), respectively. One case of TC in talocalcaneal location was missed by all three readers (Fig. 2) and all other missed cases were missed by one reader, respectively. In the control group, one case was diagnosed false-positive by two readers as calcaneonavicular TC (Fig. 3). All other false-positive diagnoses of the control group were made by one reader, respectively.

### Associated pathologies

**Fig. 4:**
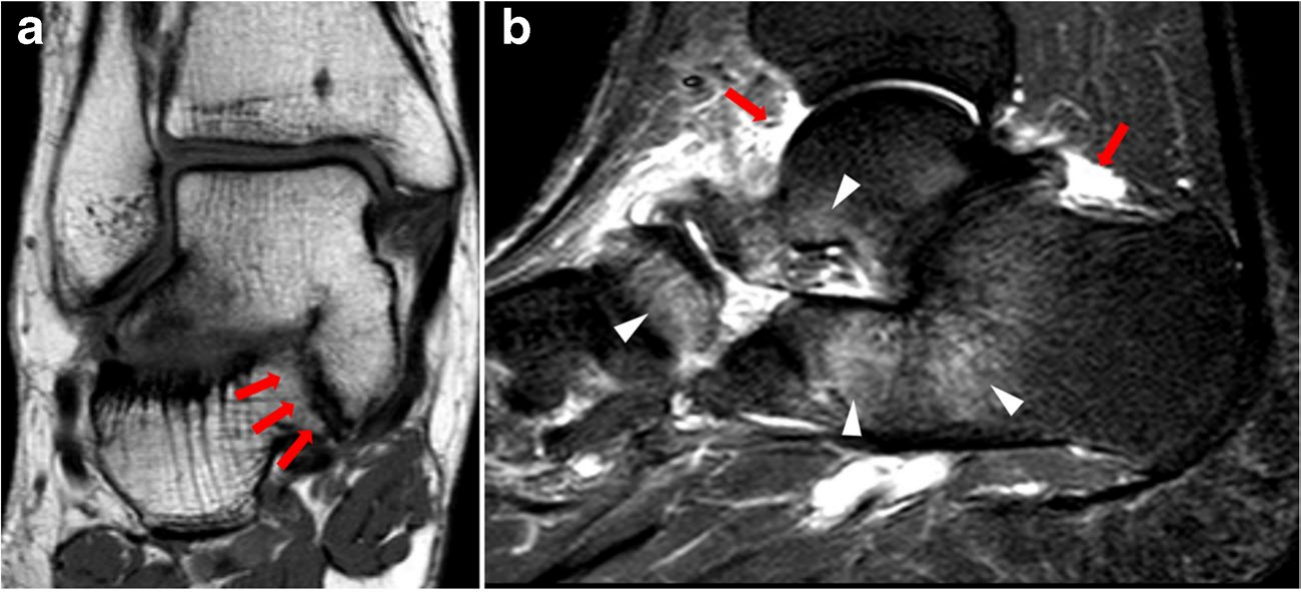
Coronal T1-weighted MR image of a 25-year-old male patient admitted for pain in the right foot diagnosed with talocalcaneal coalition in the middle facet (arrows, **a**), correctly diagnosed by all three readers. Sagittal STIR image in the same patient depicting diffuse bone marrow edema of the hindfoot (arrowheads) and joint effusion (arrows) (**b**)

**Fig. 5:**
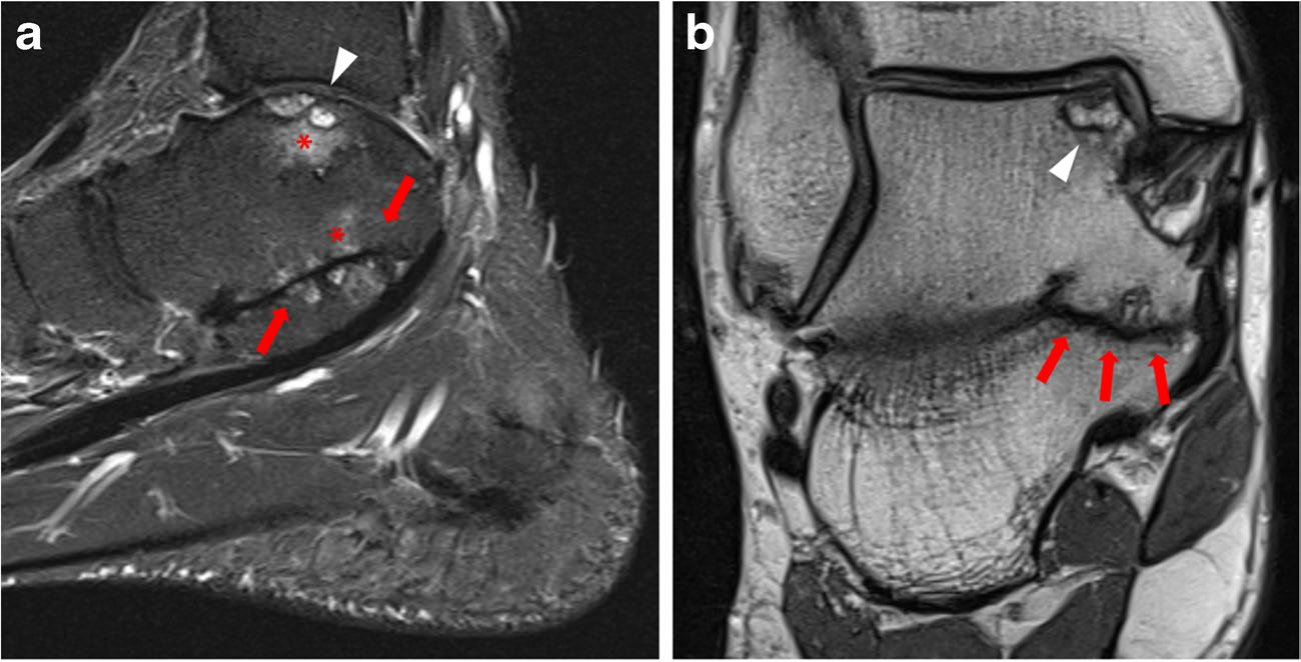
Sagittal STIR image of a 32-year-old patient with pain in the right foot diagnosed with a talocalcaneal coalition in middle and posterior location (arrows), correctly diagnosed by all three readers (**a**, **b**). Osteochondral defect (OCD) of the medial talar dome (arrowheads) along with bone marrow edema (asterisks) adjacent to the OCD and coalition as typical associated pathologies

**Fig. 6:**
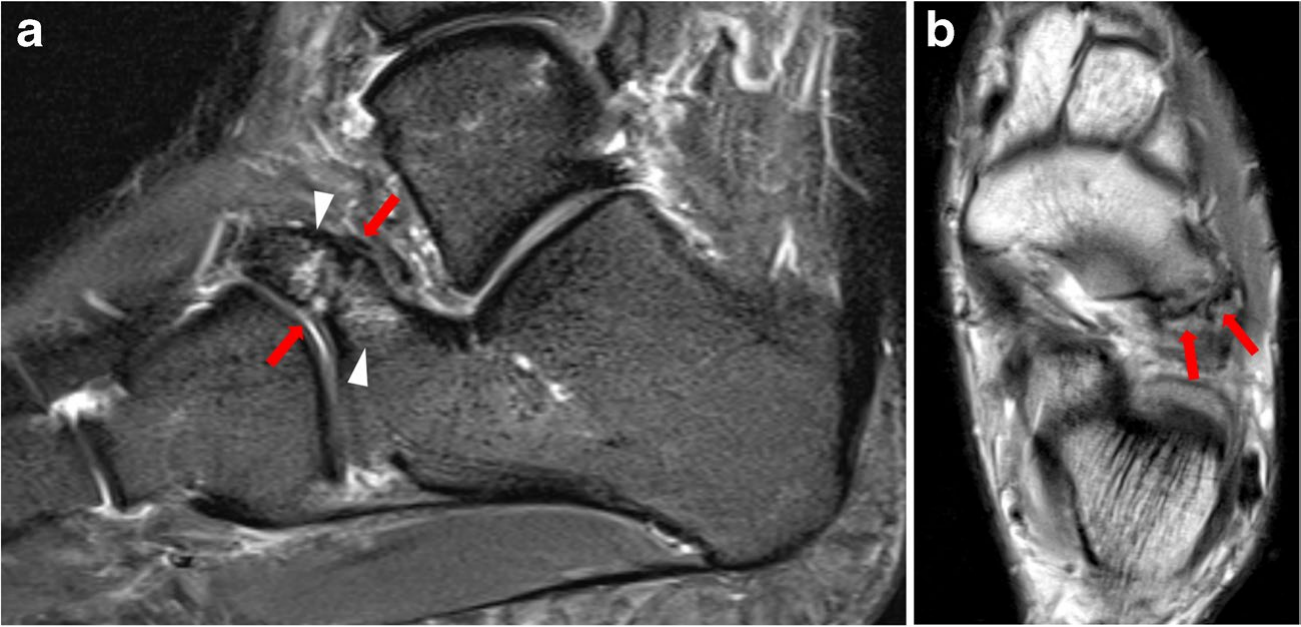
23-year-old female patient admitted with pain of the left foot and calcaneonavicular coalition, correctly diagnosed by all three readers. Calcaneonavicular coalition (arrows) with adjacent osseous marrow edema (arrowheads) is seen on sagittal STIR image and axial T1-weighted image (**a**, **b**)

Associated pathologies in the coalition group included adjacent and distant bone stress reaction (57.1% and 25.0%, respectively), joint effusion (10.7%), and OCD of the talar dome (19.6%) (Figs. 4, 5, and 6). In the control group, 64.3% of all examinations revealed bone stress reaction and 17.9% revealed joint effusion, respectively, while talar OCD was observed in 8.9% of the examinations. The number of bone stress reaction and joint effusion did not differ significantly between the groups (*p* = 0.170 and *p* = 0.228, respectively). However, the number of talar OCD differs significantly (*p* = 0.047). All characteristics of the coalition group and control group are summarized in Table 2.

In the coalition group, there was a significant association and increased likelihood between patient age and OCD (*p* = 0.03; OR = 1.060, 95% CI 1.001–1.116), but not for patient sex or anatomic location of TC (Table 4). Similarly, a significant correlation was found between talar OCD and patient age in the control group (Table 5). There was no significant correlation between adjacent or distant bone marrow edema to patient age, sex, or anatomic location of TC (*p* ≥ 0.097) (Table 4). An AALTF was found in 17.9% of patients in the coalition group and 7.1% of patients in the control group (*p* = 0.039). In the coalition group, AALTFs were observed in one case of a talocalcaneal coalition of the middle facet and in nine cases of talocalcaneal coalitions of both the middle and posterior facets.

**Table 4:** Detailed analysis of pathologies associated with tarsal coalitions. Mean and standard deviation as well as odds ratio of talar OCD, adjacent and distant bone marrow edema to age, female sex, and talocalcaneal coalition are provided. *CI*, confidence interval; *OCD*, osteochondral defect

			*p*-value	Odds ratio (95% CI)
Talar OCD	Yes (*n* = 11)	No (*n* = 45)		
Age (years)	35.9 ± 11.5	25.5 ± 13.0	0.033*	1.060 (1.001–1.116)
Sex (female; *n*, %)	7 (63.6)	20 (44.4)	0.229	2.275 (0.584–8.862)
Location (talocalcaneal; *n*, %)	5 (45.5)	33 (73.3)	0.097	0.328 (0.085–1.265)
Adjacent bone marrow edema	Yes (*n* = 30)	No (*n* = 26)		
Age (years)	27.6 ± 14.8	27.4 ± 11.6	0.939	1.002 (0.963–1.042)
Sex (female; *n*, %)	12 (40.0)	15 (57.7)	0.153	0.463 (0.160–1.339)
Location (talocalcaneal; *n*, %)	22 (73.3)	16 (61.5)	0.452	1.528 (0.505–4.622)
Distant bone marrow edema	Yes (*n* = 14)	No (*n* = 42)		
Age (years)	29.6 ± 13.2	26.8 ± 13.4	0.253	1.015 (0.971–1.062)
Sex (female; *n*, %)	9 (64.3)	18 (42.9)	0.144	2.500 (0.716–8.725)
Location (talocalcaneal; *n*, %)	10 (71.4)	28 (66.7)	0.663	1.339 (0.358–5.005)

^*^ denotes statistical significance

**Table 5:** Detailed analysis of mid- and hindfoot pathologies in the control group. Mean and standard deviation as well as odds ratio of talar OCD and bone marrow edema are provided. *CI*, confidence interval; *OCD*, osteochondral defect

			*p*-value	Odds ratio (95% CI)
Talar OCD	Yes (*n* = 10)	No (*n* = 102)		
Age (years)	36.5 ± 10.6	24.8 ± 12.5	0.018*	1.070 (1.002–1.118)
Sex (female; *n*, %)	4 (40.0)	47 (46.1)	0.713	0.780 (0.208–2.931)
Bone marrow edema	Yes (*n* = 72)	No (*n* = 40)		
Age (years)	27.9 ± 13.6	27.0 ± 12.1	0.891	1.003 (0.954–1.045)
Sex (female; *n*, %)	34 (47.2)	17 (42.5)	0.762	0.936 (0.607–1.444)

^*^ denotes statistical significance

## Discussion

Tarsal coalitions (TCs) are a common cause of considerable pain and morbidity in the foot [29, 30]. Some TC are large and these are obvious findings on diagnostic imaging, but many are more difficult to diagnose, especially the smaller ones, making TC a frequently missed diagnosis [31]. Up-to-date CT is still considered the standard modality for examining patients with clinically suspected TC [32, 33], based on studies mainly published in the 1990s [12–16]. One of the main rationales for conducting this study was that there is no current literature investigating the diagnostic performance of MRI in the diagnosis of TC, and only a few studies with small sample sizes have been published on this topic a considerable number of years ago [23–26]. In a study by Emery et al from the late 1990s, the authors state that even though MRI has a high agreement and nearly equivalent diagnostic accuracy with CT (88% for MRI vs. 94% for CT), the latter is more cost-effective and specific than MRI, especially when clinical suspicion of TC was high [23]. Although we agree with this in principle, we believe that the identification of the origin of foot pain through clinical examination is a challenge in everyday clinical practice. In our experience, except for the evaluation of osseous trauma, after initial radiographs, most patients with unclear foot pain are referred for MRI for a comprehensive evaluation of the foot, including the assessment of osseous structures, joints, and ligaments. We set out to evaluate the accuracy of MR examinations for diagnosing TC, and our study revealed an excellent cumulated sensitivity and specificity of MRI, as well as showed an almost perfect inter-reader reliability in diagnosing TC. Furthermore, different MRI field strengths (1.5 T and 3 T) were evaluated for their diagnostic accuracy. The superior performance of 3.0-T MRI observed in our study may be due to the higher spatial resolution of modern scanners, allowing visualization of smaller anatomical structures and joint abnormalities [34].

The ability of MRI to assess inflammatory changes and bone marrow edema and to visualize cartilaginous and fibrous tissue is also of great value [31], as bone marrow edema adjacent to the coalition and OCD of the talar dome were commonly observed in our study (82.1% and 19.6%, respectively). Moreover, the percentage of non-osseous TC in the present study was substantial (91.1%), which is in line with other recently published studies suggesting an increased prevalence of talar OCD and bone stress injury in patients with TC [19, 20]. Notably, the difference in the prevalence of osseous talocalcaneal coalitions in the present study (13.5%) and in a meta-analysis published by Nalaboff et al (33.3%) [5] may be due to the increased use of MRI over the past decade [34].

The association between patient age and prevalence of talar OCD was in line with epidemiologic studies, in which individuals between the ages of 33 and 45 years presented a 1.6 times higher likelihood of developing ankle OCD when compared to individuals between the ages of 20 and 32, with risk increasing by 3% for each year of age [35]. Furthermore, we observed an association between talocalcaneal coalitions and an accessory anterolateral talar facet (AALTF), which is consistent with the findings of Alqahtani et al [21]. Similar to the aforementioned study, AALTFs were observed in cases of talocalcaneal coalitions involving either the middle facet or both the posterior and middle facets. Future studies may further explore this finding, as it often escapes detection on MRI due to its small size and lack of familiarity, and the clinical implications for talocalcaneal (TC) patients with and without AALTF are currently unclear [22].

If patients with known TC are sent for preoperative evaluation, diagnostic imaging must meet certain requirements: First, the coalition has to be assessed comprehensively, which includes evaluating its dimension, depth, and composition and presence of additional coalitions [3]. Moreover, it is important to assess the extent of joint involvement, the severity of hindfoot valgus, and the existence of joint degeneration, since those parameters impact the outcome after surgery [8, 36]. The ability of MRI to meet these criteria is demonstrated in a study by Ellsworth et al. [34], in which the authors evaluated coalition size measurements in coronal PD-weighted images with excellent reproducibility, a sequence that was also part of the clinical protocol in our study. Of note, detailed TC assessment was not the primary endpoint of this study.

On the other hand, MRI might entail problems of false-positive findings, as shown by the five falsely diagnosed cases in the study. It is crucial to keep in mind that there are various anatomical variations that can be mistaken for extra-articular non-osseous coalitions. Some anatomical variations, e.g., congenital thickening of the bifurcate ligament, the posterior capsule of the middle subtalar joint, and the anterior capsule of the posterior subtalar joint or of the naviculocuboid ligament, may resemble fibrous coalitions. However, the absence of bone deformities, irregularities, and bone marrow edema indicates that the abnormality is actually an anatomical variant, rather than a TC [11, 37].

This study has several limitations. First, the retrospective design of our study might have led to a selection bias, since patients with prior surgery were excluded. Second, not all TCs were surgically correlated. Third, only talocalcaneal or calcaneonavicular coalitions and mostly non-osseous TC were identified in this study. Therefore, we cannot be certain that the sensitivity and specificity values of our study apply to coalitions in less common locations or of the osseous type. In addition, we did not perform a detailed evaluation of TC depth and length or measure the degree of hindfoot valgus. Finally, no additional information about the patient’s medical history, such as sports activities, could be obtained from the local hospital information system.

In summary, MRI is an excellent modality for diagnosing TC and can be implemented as the cross-sectional imaging method of choice in patients presenting with foot pain and suspected diagnosis of TC. The high sensitivity and specificity of MRI, coupled with its ability to identify associated pathologies, such as bone marrow edema, joint effusion, talar OCD, and adjacent accessory bones, can aid the surgeon in timely and effective clinical management of this condition. Moreover, patient care is enhanced by avoiding radiation exposure.

## Abbreviations

AALTF: Accessory anterolateral talar facet
CI: Confidence interval
CT: Computed tomography
MRI: Magnetic resonance imaging
OCD: Osteochondral defect
OR: Odds ratio
PACS: Picture Archiving and Communications System
PD: Proton density
STIR: Short tau inversion recovery
TC: Tarsal coalition

## Declarations

### Guarantor

The scientific guarantor of this publication is Reto Sutter, MD.

### Statistics and biometry

No complex statistical methods were necessary for this paper.

### Study subjects or cohorts overlap

No study subjects or cohorts have been previously reported.

### Methodology

• retrospective

• observational

• performed at one institution
