# Correction: How accurate is MRI for diagnosing tarsal coalitions? A retrospective diagnostic accuracy study

**DOI:** 10.1007/s00330-023-10531-4

**Published:** 2023-12-22

**Authors:** Adrian A. Marth, Georg C. Feuerriegel, Roy P. Marcus, Reto Sutter

**Affiliations:** 1 Department of Radiology, Balgrist University Hospital, Faculty of Medicine, University of Zurich, Forchstrasse 340, 8008 Zurich, Switzerland; 2 Swiss Center for Musculoskeletal Imaging, Balgrist Campus AG, Zurich, Switzerland

**Correction: European Radiology**

10.1007/s00330-023-10304-z

In the Discussion section of the article, paragraph beginning with "The association between patient age and prevalence… ", sentence "Furthermore, we observed an association…" the cited author "Alqahtani" is incorrectly spelled as "Alqathani".

The corrected sentence reads:

Furthermore, we observed an association between talocalcaneal coalitions and an accessory anterolateral talar facet (AALTF), which is consistent with the findings of Alqahtani et al. [21].

The original article has been corrected.

